# Fatty Acids Consumption: The Role Metabolic Aspects Involved in Obesity and Its Associated Disorders

**DOI:** 10.3390/nu9101158

**Published:** 2017-10-22

**Authors:** Priscila Silva Figueiredo, Aline Carla Inada, Gabriela Marcelino, Carla Maiara Lopes Cardozo, Karine de Cássia Freitas, Rita de Cássia Avellaneda Guimarães, Alinne Pereira de Castro, Valter Aragão do Nascimento, Priscila Aiko Hiane

**Affiliations:** 1 Post Graduate Program in Health and Development in the Central-West Region of Brazil, Federal University of Mato Grosso do Sul-UFMS, Campo Grande, MS 79079-900, Brazil; inada.aline@gmail.com (A.C.I.); gabi19ac@gmail.com (G.M.); carlinhalopescardozo@hotmail.com (C.M.L.C.); kcfreitas@gmail.com (K.d.C.F.); rita.guimares@ufms.br (R.d.C.A.G); aragao60@hotmail.com (V.A.d.N); priscila.hiane@ufms.br (P.A.H.); 2 Post-Graduate Program in Biotechnology, Catholic University Dom Bosco, Campo Grande, MS 79117-900, Brazil; alinne_castro@ucdb.br

**Keywords:** fatty acids, obesity, obesity-related metabolic dysfunction, chronic diseases

## Abstract

Obesity and its associated disorders, such as insulin resistance, dyslipidemia, metabolic inflammation, dysbiosis, and non-alcoholic hepatic steatosis, are involved in several molecular and inflammatory mechanisms that alter the metabolism. Food habit changes, such as the quality of fatty acids in the diet, are proposed to treat and prevent these disorders. Some studies demonstrated that saturated fatty acids (SFA) are considered detrimental for treating these disorders. A high fat diet rich in palmitic acid, a SFA, is associated with lower insulin sensitivity and it may also increase atherosclerosis parameters. On the other hand, a high intake of eicosapentaenoic (EPA) and docosahexaenoic (DHA) fatty acids may promote positive effects, especially on triglyceride levels and increased high-density lipoprotein (HDL) levels. Moreover, polyunsaturated fatty acids (PUFAs) and monounsaturated fatty acids (MUFAs) are effective at limiting the hepatic steatosis process through a series of biochemical events, such as reducing the markers of non-alcoholic hepatic steatosis, increasing the gene expression of lipid metabolism, decreasing lipogenic activity, and releasing adiponectin. This current review shows that the consumption of unsaturated fatty acids, MUFA, and PUFA, and especially EPA and DHA, which can be applied as food supplements, may promote effects on glucose and lipid metabolism, as well as on metabolic inflammation, gut microbiota, and hepatic metabolism.

## 1. Introduction

Obesity acts as a stressing agent both in adipose metabolism and in metabolic organs, including the liver, muscle, and pancreas, resulting in insulin resistance and type II diabetes mellitus DM II [1]. With the presence of obesity and the progressive expansion of adipocytes, the blood supply to the adipocytes decreases with ensuing hypoxia [2]. This expansion of adipocytes and hypoxia has been related to the onset of macrophage necrosis and infiltration into adipose tissue, leading to an overproduction of active metabolites called adipocytokines, such as glycerol, plasminogen activator inhibitor-1 (PAI-1), C-reactive protein (PCR), and proinflammatory mediators, including tumor necrosis factor alpha and interleukin-6 (TNFα and IL-6), and free fatty acids [3]. These changes initially result in an inflammatory process located in the adipose tissue, which expands to systemic inflammation associated with the development of obesity-related comorbidities [4].

The increased body fat observed in obesity is an increase in the number and/or size of adipocytes that are linked to metabolic and hemodynamic processes in the production of adipokines, which are responsible for causing insulin resistance and atherosclerosis, which are mediated by inflammatory cytokines [5]. Obese, but metabolically healthy individuals, have smaller adipocytes when compared to metabolically abnormal obese individuals, suggesting that hypertrophy of adipocytes is associated with the development of metabolic disorders [5]. 

**Figure 1:**
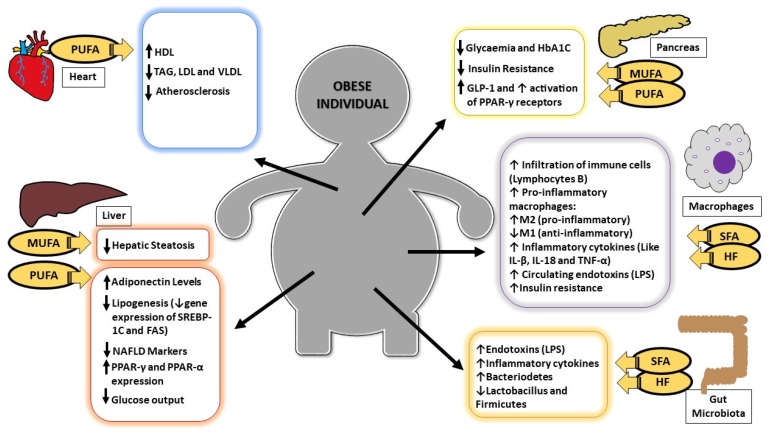
Different metabolic changes involved after consumption of different types of fatty acids: saturated (SFA), monounsaturated (MUFA), and polyunsaturated (PUFA) fatty acids. Different types of fatty acids have different effects on the major metabolic organs of the body. Diets with high levels of SFA, especially high fat (HF), modulate the inflammatory process with the infiltration of macrophages and other immunological cells, promoting higher production of type M2 macrophages, considered pro-inflammatory, with a reduction in type M1 macrophages, which are anti-inflammatory, in addition to the expression of inflammatory cytokines and circulating endotoxins, which promote insulin resistance. This inflammatory process is related to the microbiota, which also has a greater expression of inflammatory endotoxins and cytokines, as well as transitions in intestinal colonization, with increase of strains of the genus Firmicutes, and decrease of Bacteroidetes and Lactobacillus genus after consumption of HF diets rich in SFA. On the other hand, the consumption of MUFA and PUFA has positive effects on glucose metabolism, with a reduction in some parameters related to type II diabetes mellitus (DM II), such as hemoglobin A1c (HbA1c) and glycaemia, and a reduction of hepatic steatosis and related parameters. Intake of PUFA is linked to increased expression of adiponectin, an anti-inflammatory cytokine, which promotes hepatic metabolic enhancement, and reduces the risk of atherosclerosis, such as increased high density lipoprotein (HDL) and decreased triacyclglycerols (TAG). LDL; very low density lipoprotein (VLDL); glucagon-like peptide-1 (GLP-1) receptor; peroxisome proliferator-activated receptor-γ (PPAR-γ); free fatty acids (FFA); sterol regulatory element-binding protein -1C (SREBP-1C); Non-alcoholic fatty liver disease (NAFLD); interleukin (IL); tumor necrosis factor (TNF); lipopolyssacharide (LPS); high fat (HF).

With obesity, immune cells display phenotypic changes according to the type of dietary fatty acids, causing a change in the M2 macrophage, which has anti-inflammatory properties on M1 macrophages, which have pro-inflammatory properties. The consumption of saturated fatty acids (SFA) activate M1 genes that stimulate -α and IL-6 TNF production, whereas monounsaturated fatty acids (MUFA) activate the M2 genes related to the expression of Arginase-1 and interleukin-10, which are cytokines with anti-inflammatory action [6], as illustrated in Figure 1.

At the end of the 1950s, the different types of fatty acids ingested in the diet were thought to influence glucose homeostasis. Many decades later, new in vitro and in vivo studies maintained this hypothesis, pointing to different influences according to chain length and the number of double bonds in fatty acids, altering sensitivity, pro- or anti-lipotoxic action, and insulinotropic influence [7,8].

In order to control this inflammatory process and the obesity comorbidities, some strategies are used, such as changes in diet, which include the reduction of SFA sources and an increase in the consumption of MUFA and polyunsaturated fatty acids (PUFA), which are associated with cardiovascular protection, acting on atherosclerosis [9,10,11].

Mammals are able to desaturate fatty acids at positions Δ5, Δ6, and Δ9 [12]. The latter desaturase is called stearoyl-CoA desaturase (SCD-1) and it converts SFAs, such as stearic acid (18:0), into oleic acid, a MUFA; while Δ5 and Δ6 desaturases are required for long-chain PUFA desaturation. These kind of PUFAs are classified as omega-3 (ω-3) and omega-6 (ω-6) [13]. Araquidonic acid (AA) is the principal ω-6 fatty acid, whereas eicosapentaenoic (EPA) and docosahexaenoic (DHA) fatty acids are the main ω-3 FAs [14]. These FAs are defined as essential fatty acids because they cannot be synthesized in human cells, and therefore must be obtained from dietary linoleic acid (C18:2 n-6) and α-linolenic acid (C18:3 n-3) [15].

Considering the importance and the effects of fatty acid intake, the global problem of obesity, and the risk factors associated with obesity and chronic diseases, FAs are usually a component of nutrition educational programs and those individuals receive guidance for lifestyle changes. For many years, highlighting the consumption pattern of lipid content and quality in the diet, aiming at a reduction in the consumption of saturated fatty acids, was a key component of an obesity-targeted diet [16,17]. Because some lipid sources of saturated fatty acids have shown positive results, as in the case of coconut oil, extensive discussion has been generated about the quality of lipids that should be used in the diet. In addition, other measures related to lipid ingestion, proposed in the treatment of chronic diseases, should be considered, and deserves further elucidation to avoid such controversies [18].

The objective of this review is to determine the metabolic effect related to the mechanism of actions of the different types of fatty acids, including saturated, monounsaturated, and polyunsaturated acids, in obesity and its related disorders, (i.e., insulin resistance, dyslipidemia, inflammation, non-alcoholic fatty liver, and intestinal microbiota) through the compilation of several scientific papers published in the last five years.

## 2. Insulin Resistance and Associated Comorbidities

The main factors responsible for the development of type 2 diabetes mellitus (DM II) are the high production of hepatic glucose, impaired insulin secretion, and insulin resistance, which is common in obesity [19]. During DM II evolution, adipocytes become resistant to the anti-lipolytic activity of insulin, which leads to increased concentrations of free fatty acids in both the fed and postprandial states. This situation worsens considerably as the function of beta cells is impaired and insulin secretion decreases [20]. 

Dietary fatty acids play an important role in cell membranes and insulin sensitivity, interfering with the metabolic control of diabetes. Observational studies show a strong association between diets with a high amount of SFAs, being mainly palmitic acid, and a small amount of PUFA, with insulin resistance (Figure 1) [21]. Hypercaloric diets, especially hyperlipidic ones, are related to the induction of insulin resistance [22]. 

An excess of nutrient intake also directly regulates tumor cell growth, and saturated fatty acids are more associated with this inducement of proliferation than unsaturated fatty acids, which promote apoptosis [23]. A diet low in saturated fatty acids is recommended for the treatment of DM II. Among the SFAs, palmitic acid (16:0) is believed to be responsible for the damage caused to β-pancreatic cell function and to insulin resistance [24].

In a recent study [22], a hypercaloric and hyperlipidic diet in the induction of insulin resistance in humans was developed and tested. The effects of a diet rich in saturated fatty acids after 24 h included increases in glycaemia, insulin, and HDL-cholesterol levels when compared to a normocaloric and normolipid diet, whose lipid composition corresponded to 25% of the total calories, being 12% MUFA, 8% PUFA, and 5% SFA (Table 1). The negative effect of the SFAs was suggested to be greater when associated with a hypercaloric diet (>27 kcal/kg), since the SFAs probably enter the cell membranes by altering the insulin receptors and their secretion, resulting in insulin resistance [25].

In a study by Crochemore et al. [26] (Table 1), women with DM II and hypertension had no statistically significant difference among the groups that received supplementation of 1.5 g and 2.5 g fish oil, with 21.9% EPA and 14.1% DHA, respectively, and a control group, for glucose, glycated hemoglobin, insulin, or homeostasis model assessment-estimated insulin resistance (HOMA-IR), regardless of the dosage. The doses of fish oil and the duration of the study, at only 30 days, were potentially inadequate to note a significant difference in this population, which also had a hypertension frame. Additionally, despite the absence of a statistical difference, the groups supplemented with fish oil presented a trend in HOMA-IR improvement, which decreased 21.4% and 35.7%, respectively, in both the supplemented groups when compared to the baseline.

Another parameter used is Hemoglobin A1c, which reflects the cumulative changes that occur over several weeks to months, so the duration of treatment may be particularly important for this biomarker. Glycation refers to the non-enzymatic reaction of reducing sugars with primary amino groups’ Schiff bases that undergo an Amadori rearrangement, which is well-studied for proteins and peptides. Glycation sites derived from glucose have been reported and characterized by many proteins in the last decades, with the glycated N-terminal hemoglobin A (HbA1c) being a well-established biomarker to diagnose and control diabetes [32]. Müllner et al. [27] showed a decrease in this parameter after implementing a diet including 300 g of vegetables and 25 mL of PUFA-rich plant oil daily. Additionally, a significant decrease in hemoglobin A1c occurred during the eight-week test with the fish oil group, where each capsule had 3.58 g of EPA and 2.44 g of DHA [28], demonstrating the positive effects on glycated hemoglobin after the intake of polyunsaturated sources, whether of vegetable or marine origin.

Another study with DM II subjects did not show significant improvements in a large part of the glucose homeostasis parameters. The ALA group had improved insulin sensitivity, which may be associated with the greater increase in adiponectin levels also evaluated in this study, which has an inverse correlation with HOMA-IR, reinforcing the positive effect of ALA supplementation on IR [29]. Patients with DM II exhibit lower postprandial glucagon-like peptide-1 (GLP-1) responses as compared to healthy individuals [33]. GLP-1 is secreted from intestinal endocrine cells in response to nutrient intake and plays several different roles in metabolic homeostasis after its absorption [33].

The supplementation of Conjugated Linoleic Acid (CLA) resulted in improvements in obese children after four months of intervention, combined with lifestyle changes for both the children and their parents. Garibay-Nieto et al. [31] implemented a program consisting of monthly visits that included a one-hour structured physical activity session, followed by a psychoeducational group session, with consultations with nutritionists, and supplementation of CLA (3 g/day) or placebo (1 g/day) for obese children three times a day for 16 weeks, which resulted in insulin reductions (µU/mL), fasting insulinemia, and HOMA-IR in the CLA group.

CLA represents a group of positional and geometric isomers of linoleic acid (18:2n-6), whose predominant form is cis-9, trans-11 isomers, and due to its high composition of PUFA, promotes benefits in the membrane phospholipid composition, thus improving its fluidity. Another possible mechanism for the antidiabetic effects of CLA supplementation is the activation of peroxisome proliferator-activated receptor-γ (PPAR-γ) receptors [34], which participate in lipid homeostasis and are predominantly expressed in adipose tissue. With the activation of PPAR-γ, CLA also increases the gene expression of adiponectin and thus may affect glucose metabolism and insulin sensitivity [35].

Furthermore, some in vivo and in vitro studies presented positive results after the use of unsaturated sources, especially the n-3 series, and with MUFA, basically oleic fatty acid, which are demonstrated in Table 2. An in vitro study [36] demonstrated that oleate did not induce insulin resistance in cardiovascular cells, such as cardiomyocyte, vascular smooth muscle cells, or endothelial cells, which are otherwise palmitate induced, showing the beneficial cardiovascular effects in relation to insulin signaling with oleate. Previous studies showed this induction of resistance insulin with palmitate also being observed in other tissues, such as adipocytes and skeletal muscle [37,38]. Oleate was able to prevent insulin resistance in the myotubes through the activation of PI3K and a mechanism dependent on amp-activated protein kinase (AMPK) [36]. 

Malinska et al. [39] found an increase in insulin sensitivity in hypertriglyceridemia-induced dyslipidemia rats fed a high sucrose diet, supplemented with either sunflower oil or Conjugated Linoleic Acid (CLA) (2 g/100 g diet), for eight weeks. CLA possibly present anti-diabetic effects related to the activation of PPAR-γ receptors [34].

The MUFA intake also showed positive results on the glucose parameters, with a statistically significant reduction in the HbA1c levels [30]. A high-MUFA diet also showed an improvement in insulin resistance when compared to a high-SFA diet in mice, with significantly lower fasting glucose and insulin concentrations and attenuated insulin secretion, in response to glucose challenge. These challenges included intraperitoneal glucose, insulin tolerance testing, and insulin secretion response in overnight fasted mice after intraperitoneal injection with 1.5 g/kg glucose [40]. Carbohydrates and fatty acid components of a meal can directly influence postprandial GLP-1 responses [33]. MUFAs seem to be powerful stimulators of GLP-1 secretion, both in the enterocytes cultured from mice in vivo fand in Zucker rats that were genetically obese [41,42].

The liver plays a unique role in regulating glucose homeostasis by maintaining blood glucose concentration within a normal range. However, impaired insulin action in the liver leads to insulin resistance, characterized by an impaired insulin capacity to inhibit glucose production. Thus, insulin resistance in the liver, which is the reduced sensitivity to insulin in the liver, causes gluconeogenesis and hyperglycemia. As a result of insulin resistance, adipocytes increase the release of free fatty acids (FFA) in the circulatory system [44].

A study with a diet-induced IR rat model [43] showed a significant reduction in HOMA-IR after supplementation of 12 g per 100 g for 12 weeks with fish oil, when compared to the n-6 PUFA and MUFA groups (Table 2). The evidence indicates positive effects on insulin resistance and glycemic metabolism with consumption of ω-3 PUFA, which can be explained by the alteration in serum fatty acid composition, which influences the membrane fluidity with the ingestion of these polyunsaturated sources, allowing for greater insulin binding. Subsequent events, such as aggregation, internalization of the insulin-receptor complex, and the movement of the glucose transporter to the cell membrane, could be facilitated by changes in membrane fluidity [45]. 

The evidence indicates that the composition of dietary fatty acid intake can change the fatty acid composition of the cell membrane, thereby affecting insulin sensitivity [46]. A diet rich in unsaturated fatty acids, present in oilseeds, plays an important role in the prevention of insulin resistance, increasing insulin affinity for the receptors [47]. Furthermore, the composition of oilseeds with high magnesium [48,49], high fiber content [50,51], and low glycemic index [52,53] have also been linked to a lower risk of diabetes.

In addition to the previously mentioned factors associated with glucose metabolism and insulin resistance, the metabolism of this comorbidity is known to be complex, being associated with cardiovascular diseases (CVD). CVD mortality has been strongly linked to the prevalence of diabetes, in which CVD is considered an important risk factor for DM and vice versa. The metabolism involved in the CVD should be addressed with the consumption of different lipid sources, accessing the different influences in the comorbidities linked to obesity [54].

## 3. Dyslipidemias

Dyslipidemia is characterized by the change in lipid concentrations in the bloodstream with the accumulation of one or more classes of lipoproteins [55]. In this case, this process is called hypercholesterolemia, which is the accumulation of low density lipoprotein (LDL) in the plasma by alterations in the genes of the LDL receptor (LDLR), or apolipoprotein B-100, which is considered as one of the most important components of atherogenic lipoproteins [55]. Hypertriglyceridemia is the result of chylomicron condensation of very low density lipoprotein (VLDL), or both lipoproteins in the plasma, with a decrease in high density lipoprotein (HDL) [56]. These condensations are as a result of lipoprotein lipase (LPL), which is responsible for promoting triglyceride breakdown with apolipoprotein A5 (APOA5) mutations, and the inhibition of LPL and hepatic lipase by apolipoprotein C-III (APOC3), contributing to the triglycerides concentration increase [57].

The atherogenic process occurs when Lymphocytes T migrates to the intima layer of the arteries by proinflammatory cytokines, producing macrophages by monocytes and increasing the LDL levels, resulting in atherosclerotic plaques [58]. These plaques originate in the intima and media layers of medium and large arteries, and are thus the principal factor related to CVD [59]. 

Some types of treatment and prevention methods are available to address dyslipidemia and atherosclerosis, such as specific medicines, as well as the adoption of a healthy lifestyle, which includes an improvement in eating habits [60]. One of the reasons for changing these habits is to improve the quality of lipids consumed in the diet, involving recommendations to reduce SFAs, which are associated with the dyslidemia process because they cause an increase in LDL, which is due to a reduction in the production and activity of the LDLR gene, related to alter the hepatic metabolic processes and fatty acid biosynthesis [61]. 

Besides that, recommendations are proposed to increase the ingestion of PUFA sources in detrimental of SFA reduce. The n-3 PUFA consumption operates on the reduction of plasma triacyclglycerols (TAG), VLDL, and apolipoprotein B-100 (APOB-100) [62,63]. A multinacional study (Table 3) with individuals at least 18 years of age with average serum TG concentrations >500 mg/dL but <2000 mg/dL at screening (1 and 2 weeks before random assignment) who were either untreated for dyslipidemia or were using a stable (for at least 6 weeks before the first qualifying lipid measurement) dosage of a statin, CAI, or their combination. The individuals were divided into four groups, which one of them was a control group with 4g/day of olive oil supplemented. There were three groups that receive the supplementation of fish oil (EPA + DHA) in capsules of 2, 3, and 4 g/day. All of the groups that receive the fish oil presented decreasing values for TG, non-HDL, LDL, VLDL and ApoB-100 from baseline [63]. n-3 PUFA acts on ApoB-100 by inhibiting its synthesis and, consequently, decreasing the plasma concentration of all lipoproteins composed with Apo-B, especially VLDL and LDL [64]. DHA also presents an important mechanism in the signaling pathway, inhibiting the delta-6 desaturase, an indispensable enzyme that produces gamma linolenic and dihomo-gamma-linolenic acids from linolenic acid, to the detriment of the production of araquidonic acid (AA). In addition, DHA acts to improve cellular membrane fluids, which enables the flexibility of the arteries, aiding in the removal of lipids, as well as possible inflammatory agents, that are deposited in the membrane [63].

Mattos et al. [65] performed a randomized study with administration of two fish oil capsules per day in hemodialysis patients for 12 weeks. The results did not demonstrate differences between the groups that received n-3-PUFA and the control group that received a placebo. This may have occurred because hemodialysis patients are have a higher propensity of developing CVD.

In another study [68], after the use of flaxseed oil, Echium seed oil, and microalgae oil, which is another source of n-3-PUFA with high DHA, changes in LDL levels were not seen, however, they observed an increase in HDL levels. This result corroborates with a study by Wang et al. [67], who observed that individuals that consumed four capsules of fish oil, containing EPA and DHA, for six weeks showed no differences in total cholesterol (TC) and LDL, whereas TAG levels were reduced.

The following molecular mechanisms responsible for the reduction of serum TAGs, after EPA and DHA ingestion, have been proposed to create this beneficial effect: the decreased expression of sterol regulatory element-binding protein-1c (SREBP-1c) may be one of the factors responsible for the reduced secretion of VLDL-TAG, or the increased mitochondrial oxidation rates, or peroxisome that reduces the substrate for TAG synthesis [69]. 

The expression of decreased SREBP-1c can be mediated by the inhibition of liver X-receptor binding (LXR) to the LXR/retinoid X receptor. The increase in peroxisomal oxidation rates may result in the increase of peroxisome proliferator-activated receptor-α (PPAR-α) on the expression of the acyl-coenzyme A oxidase gene. In addition, the reduction in the activity of the enzymes that perform TAG synthesis decreases the distribution of non-esterified fatty acids from adipose tissue and decreases the availability of apoprotein B, which potentially results in a lower release of VLDL-TAG. On the periphery, increased lipoprotein lipase activity may lead to increased clearance of VLDL-TAG, possibly due to increased PPAR-γ and/or PPAR-α gene expression [69]. Additionally, EPA and DHA fatty acids are capable of preventing the synthesis and the secretion of hepatic VLDL and TAG, increasing the TAG clearance by chylomicrons and VLDL particles. Furthermore, both EPA and DHA are preferably diverted for phospholipid synthesis paths, whereas other fatty acids, such as oleic acid, are generally incorporated into TAG [70].

Furthermore, n-3 PUFAs promoted an increase in HDL in the studies that used EPA and DHA. This mechanism may improve the atherosclerosis protection due to the fact that n-3-PUFAs act in reverse cholesterol trafficking [71]. This involves of the transportation of cholesterol molecules present in high concentration in the tissue, back to the liver to be eliminated by bile in feces, improving the endothelial dysfunction and promoting antioxidants and anti-inflammatory effects [71].

Considering that animal models are extremely important for elucidating the etiology of diseases in humans, having an integrated view of disorders mechanisms [72], there are some in vivo studies in Table 4 on dyslipidemia. A treatment with rats demonstrated that a high amount of SFAs in the diet during a 30-day study resulted in increased levels of total cholesterol (TC), TGA, LDL, and VLDL in the bloodstream, as shown in Table 4 [73]. On the other hand, after 180 days of diet, TC, HDL, and LDL serum levels increased, while VLDL and TGA levels diminished. The data showed that a diet rich in SFA exerted a hyperlipidemic effect only on the 30th day, but a long-term diet displayed beneficial effects on hyperlipidemia (Table 4) [73], which could be a consequence of the decrease in apolipoprotein synthesis and the formation of VLDL in the liver [74,75].

SFA and n-6 PUFA showed a negative effect on TC, HDL, and TAG levels, which increased in relation to the groups that received flaxseed oil, including alfa-linolenic fatty acid (ALA) and SFO (a combination of sesame and flaxseed oil; n-3 and n-6 PUFA), in wistar rats after 65 days of treatment with a standard diet poor in lipid sources, at 7% fat [76]. The SFO groups also demonstrated an improvement in non-HDL and LDL levels when compared to others (Table 4). This could be explained by the proportion of n-6 to n-3 PUFA in this diet, which was 1:1, which is considered the best proportion of essential fatty acids associated with cardioprotective effects [77]. According to another study with pigs, a diet with the proportions of n-6:n-3 from 1:1 up to 5:1 better assisted in the use and absorption of fatty acids, also promoting an anti-inflammatory action [78].

For MUFA, Macri et al. [79] showed that sunflower oil consumption resulted in an increase in visceral fat depots and liver weight, due to cholesterol esters that are supplied by oleic acid from the diet, contributing to cholesterol oleate synthesis in the liver and to the secretion of lipoproteins that possess ApoB, like LDL. On the other hand, olive oil led to a reduction in TC and LDL, which contributes to the anti-atherogenic effect.

In a similar study performed by Alsina et al. [80], fish-oil supplemented high-oleic-sunflower oil group (HOSO-F) supplementation diminished mesenteric, epidydimal, and perirenal fats, which become visceral fat deposits. The group that received HOSO supplemented with fish oil or phytosterols (F) displayed an improvement in lipid serum levels and fat deposits. Moreover, supplementation with F resulted in the inhibition of cholesterol absorption in the gastrointestinal tract. During the digestive process, cholesterol in the diet is solubilized by bile acids, incorporated in mixed micelles, and absorbed in enterocytes through the Niemann-Pick C1 Like-1 transporter [82]. F are metabolized in the same way as cholesterol involved in dynamic competition. Finally, F dislocate cholesterol from micelles and the micelles are eliminated by feces [83].

A study [81] with 96 wistar rats in their experimental study, separated them into four groups according to weight and the concentration of TG in plasma and cholesterol. The standard diet was supplemented with 15% of different sources of fat, with the SFA group with bovine serum, used as control; MUFA, having as source the olive oil; PUFA n-3 using fish oil; and, PUFA n-6 with safflower oil. TG levels showed a significant reduction in the first day of supplementation of MUFA and PUFA-6, which when compared to groups SFA and PUFA, n-3 had higher statistical significance. However, after three weeks of supplementation, the MUFA and n-6 PUFA groups returned to TG levels prior to treatment, increasing. Regarding long-term treatment, there was a reduction in the TG levels only in PUFA-3 supplementation. In this study, plasma cholesterol did not show changes in supplementation at both experimental times, a short- and long-term reduction was observed only in animals supplemented with PUFA-3. This TG reduction was accompained by a decrease of activities of the lipogenic enzymes acetyl-CoA carboxylase (ACC) and fatty acid synthase (FAS), as well as a decreased activity of the citrate carrier (CIC), a mitochondrial protein linked to lipogenesis [81].

It is believed that inhibition of de novo lipogenesis (DNL) may be a viable approach to treating obesity-related disorders, especially in rodents. The decreased DNL is related to reducing the amount of synthesized fatty acids that enter in the pathway of esterification and, resulting in minor’s levels of TG for VLDL assembly [81,84]. The hypotriglyceridemic effect of PUFA is partly caused by the reduced activities of liver lipogenic enzymes and by increased β-oxidation, consistent with increased mitochondrial as compared to peroxisomal oxidation [85]. 

The therapeutic potential of n-PUFA is important because it mediates the biological processes, such as eicosanoid production [86], which creates signaling molecules including leukotrienes, prostaglandins, thromboxane, and prostacyclins. These molecules are responsible for different cellular functions such as chemotaxis (blood cell migration), platelet aggregation, and cellular growth, demonstrating that the type of fatty acid consumed influences inflammation [87].

## 4. Inflammatory Process and Intestinal Microbiota

Low-grade chronic inflammation contributes to the inflammatory state in adipose tissue of obese individuals, mediated by innate immunity that leads to the production of proinflammatory cytokines, such as TNF-α, IL-1, IL-6, and IL-1β. The excess of adipose tissue favors the exaggerated release of free fatty acids through the action of catecholamines. This process inhibits the capture of glucose, generating a state of hyperglycemia that may cause hyperinsulinemia. The inflammatory process is characterized by the infiltration of macrophages and lymphocytes into adipose tissue and even into other peripheral organs. It results in an imbalance responsible for increasing the production of inflammatory cytokines that contribute to the onset of other metabolic dysfunctions, such as insulin resistance, since they may inhibit signaling or even insulin receptors [88,89,90]. 

Changes in diet quality may then improve inflammatory markers, as observed in 22 obese children and adolescents, with a body mass index (BMI) beyond the 95th percentile for age and sex, before and after the qualitative change in their food consumption [90]. The researchers relied on therapeutic protocols, suggesting a lower consumption of foods high in lipids and sugars and an increase in food sources of fiber, having only quantitative control over the portion sizes consumed, were beneficial. As a result, obese individuals (Table 5), when evaluated prior to intervention, had high values for various inflammatory cytokines, such as IL-1β and IL-18, which are associated with inflammatory and autoimmune disorders. INF-γ, IL-12A, IL-6, and TNF-α also decreased after 18 months of intervention. They also observed a decrease in lipopolyssacharide (LPS) and CD14 even without a significant decrease in BMI [90]. 

Another study [91] (Table 5) evaluated four types of diets with different FA, and found that postprandial endotoxin is influenced by the FA composition of the diet and not by the fat content itself. The results indicated that subjects consuming n-3 PUFA meals decreased their serum endotoxin levels, unlike those who consumed the n-6 PUFA meals, which increased these levels, but the inflammatory markers themselves did not show any changes. This was justified by the small number of participants, considered healthy, and used only a single meal as a source of evaluation. Nonetheless, lower endotoxin levels agree with Simopoulos [92], who considered n-6 PUFA as being responsible for the increase in cellular triglycerides and for the permeability of membrane, which can lead to the accumulation of adipose tissue fat, which is highly pro-inflammatory, and has pro-thrombotic and pro-adipogenic roles. Therefore, the proportion of consumption in relation to the n-6 PUFA and n-3 PUFA rate should be balanced, with the n-3 PUFA consumption being higher to preserve its protective role for metabolic disorders, especially in relation to the inflammatory state.

In another report [93], the effects of HF diets with different concentrations of palmitic acid and oleic acid on metabolism of obese adults were studied over three weeks of treatment. There was a diet with high content of palmitic acid (HPA) and moderate in oleic acid (OA) (fat, 40.4% kcal; PA, 16.0% kcal; OA, 16.2% kcal), and a diet low in PA and high in OA (HOA) (fat, 40.1% kcal; PA, 2.4% kcal; OA, 28.8% kcal). The HPA diet resulted in a decrease in IL-1B, an inflammatory marker, when compared to the control diet with 15.9% OA. This diet also resulted in a decrease in TNF-α, IL-18, and IL-10 levels. On the other hand, the HOA diet showed an increase in these same inflammatory markers, showing that the uneven proportion between these two fatty acids (FA) in the diet may increase inflammatory cytokines, thus triggering this process.

Haro et al. [94] aimed to study the changes in microbiota after one year’s consumption of a Mediterranean diet (MD) or a low-fat, high-complex carbohydrate diet (LFHCC diet) in an obese population, within the Coronary Diet Intervention With Olive Oil and Cardiovascular Prevention (CORDIOPREV) study, an ongoing prospective, randomized, opened, controlled trial in patients with coronary heart disease. The participants were randomized to receive the MD (35% fat, 22% monounsaturated) and the LFHCC diet (28% fat, 12% monounsaturated). The MD diet consumption and LFHCC diet increases the abundance of the Roseburia genus and F. prausnitzii, respectively. Roseburia is related to produce an inhibitory substance against *Bacillus subtilis* (Hatziioanou), suggesting MD induce some changes in the microbiota mediated by the antimicrobial effect of this genera, which modifies the microbial population in the colon. On the other hand, LFHCC consumption increased the abundance of another diabetes-protective bacterial species, F. prausnitzii (found to be low in patients with DM II). These two changes after MD and LFHCC diets could have a protective influence for the prevention of T2D, suggested by the findings of an improvement in insulin sensitivity after the consumption of the both diets.

A randomized, controlled, double-blind, crossover clinical trial study with 33 hypercholesterolemic volunteers, aged 35–80 years was carried out [96]. Participants ingested 25 mL/day for 3 weeks, preceded by 2-weekwashout periods, three raw virgin olive oils differing in the concentration and origin of phenolic compounds (PC): (1) a virgin olive oil (OO) naturally containing 80 mg of PC/kg, (VOO), (2) a PC enriched virgin olive oil containing 500 mg PC/kg, from OO (FVOO), and (3) a PC-enriched virgin olive oil containing a mixture of 500 mg PC/kg from OO and thyme 1:1 (FVOOT). The OO group did not present changes in microbiota, whereas the FVOOT group presented an increase in the group of Bifidobacteria, Parascardovia denticolens and Roseburia.

Another study evaluated the effects of PUFA n-3 from sardine. The patients with DM2 were randomized to follow either a type 2 diabetes standard diet (control group: CG), or a standard diet enriched with 100 g of sardines 5 days a week (sardine group: SG), which represented a dose of EPA + DHA of 3 g per day, for 6 months. There was a decrease in phylum Firmicutes in both groups and in the Firmicutes/Bacteroidetes ratio in the SG group over time, and a decrease in Bacteroidetes/Prevotella ratio in CG group. The SG presented an increase in adiponectin levels, whereas CG group showed an increase of in TNF-α [97].

Some volunteers at increased Metabolic Syndrome (MetS) [95] risk followed five diets: high saturated fat diet (HS; saturated fatty acids, SFA); high monounsaturated fat (MUFA)/high glycemic index (GI) (HM/HGI); high MUFA/low GI (HM/LGI); high carbohydrate (CHO)/high GI (HC/HGI); and, high CHO/low GI (HC/LGI) for 24 weeks. The reduction of dietary fat intake and increasing dietary carbohydrate consumption increased both faecal *Bacteroides* and *Bifidobacterium* spp., which are linked to improve body energy regulation and reduced risk factors of MetS. Besides that, increased Bacteroides numbers after the HC/HGI diet were directly and significantly correlated with a modest decrease in body weight, waist circumference and body mass index (BMI). An increase in Bifidobacterium was also observed on both low-fat high-CHO diets, and also had showed a modest increase in Atopobium numbers, both within the Actinobacteria phylum, which are dominant members of the human gastrointestinal microbiota, and are considered important degraders of carbohydrate. These bacteria’s growth may have been stimulated by the increased bioavailability of dietary carbohydrate. 

Moya-Pérez et al. [89] (Table 6) showed that high fat (HF) diets are responsible for increasing the infiltration of lymphocytes B in rats, which are the first cells in the immune system to be recruited from adipose tissue after administration of these diets. Lymphocytes B also increase insulin sensitivity by activating T cells and increasing the release of proinflammatory macrophages, thus contributing to the inflammation process with the production of IL-8 and interferon-γ (IFN-γ) cytokines. In another study, Masi et al. [98] evaluated the effect of high sugar (HS), HF, and HS and HF diets on mice over a period of eight weeks. The caloric intake from the HF groups was lower. All three diets increased the size of adipocytes and hepatocytes when compared to the control group, and only the HS and HF diet showed a significant increase in proinflammatory cytokines (IL-6 and IL-1β), showing that the increase in the consumption of sugar increases the lipogenesis, promoting the storage of the triglycerides. 

This increasement in adipocyte size was also observed when high fat diets (HF) were administered to rats [5], in which 51% of the energy was derived from fats, and they observed that an increase in adipocyte size occurred, and as a consequence, an increase in inflammatory cytokines (NF-γ, IL-6 e TNF-α) was observed, as shown in Table 6. Caër et al. [88] reported that adipocytes are exposed to the effects of inflammatory factors, hormones, and even pollutants. This alters their metabolic capacity and cellular functions through the action of IL-1β, IL-17, and TNF-α, and can lead to a greater accumulation of fat. TNF-α, for example, acts on the lipolytic pathway of these adipocytes, maintaining the fat mass, restricting excess adipocyte production and accumulating lipids.

The beneficial role of fiber, in a hyperlipidic diet on inflammatory markers, was also verified in Moran-Ramos et al. [99] (Table 6). They evaluated the effects of Nopal fibers, a medicinally used plant in Mexico, and found a decrease in adipocytes size and Il-6 levels, when administered as part of a HF diet over a six-week period. These fibers were able to alter the intestinal microbiota and increase fermentation rates, showing their role in preventing intestinal inflammation in being able to increase the beneficial forms of microbial diversity. 

The administration of n-3 PUFAs have also shown beneficial anti-inflammatory action. The main metabolites of this essential fatty acid are EPA and DHA, considered polyunsaturated long chain fatty acids, with the first double bond in the third carbon of its chain. It is found in large quantities in fish, such as tuna and salmon [104]. Some studies point to this protective factor following the administration of fish oil, with a decrease in the production of TNF-α, IL-1β, and IL-6 by monocytes that were stimulated by endotoxins or mononuclear cells (Table 6). These fatty acids (FA) are responsible for inducing a change in inflammation activity through their incorporation into the phospholipids of inflammatory cells that cause a greater membrane fluidity, modifying the lipid derivatives that will be formed. Thus, it has effect on various anti-inflammatory responses, such as the production of eicosanoids and cytokines, and also on various types of cells, such as monocytes and macrophages [104]. 

The isolated use of EPA with 1% supplementation in HF given to C57BL/6J mice for 16 weeks was beneficial in the reduction of total cholesterol, and in the reduction of adipocyte size. In addition, it reduced plasma levels of leptin by approximately 60%, considered a pro-inflammatory cytokine [105]. Besides that, another study showed that EPA ameliorates HF-diet effects in mice and cultured adipocytes, which EPA increased the oxygen consumption and fatty acid oxidation and reducing adipocyte size, adipogenesis, and adipose tissue inflammation, independent of obesity [106].

A hyperlipidic diet, associated with the use of antibiotics, can lead to intestinal dysbiosis. Dysbiosis is an imbalance that causes an increase in bacterial growth, production of toxins, and an increase in intestinal permeability, affecting the transient microbiota, thus causing some disorders [107]. In addition, individuals predisposed to obesity may be present with intestinal microbial communities that promote the storage of energy, different than in lean individuals. Different compositions and even administration of strains, such as bifidiobacteria, may influence the production of proinflammatory cytokines [108]. Moya-Pérez et al. [89] administered strains of *B. pseudocatenalatum* in both a placebo and an obese group, with a HF diet over a six-week period. These strains were able to decrease inflammatory markers such as TNF-α, IL-6 and INF-γ in the HF group, which also resulted in a weight reduction. They suggested that the reduction of INF-γ occurred due to the action of the bacteria regardless of the type of diet offered.

The gastrointestinal bacteria, such as *Bacteroides thetaiotaomicron*, are responsible for the digestion of fibers. They produce short chain fatty acids (SCFA), such as butyrate, propionate, and acetate, which serve as energy substrates for other bacteria [108]. Butyrate affects inflammatory mediators since they are able to inhibit the expression of pro-inflammatory cytokines by inhibiting nuclear factor κB (NF-κB). They may also cause changes in the intestinal epithelium, leading to increased intestinal permeability. Acetate is the main SCFA in the colon and acts as a substrate for cholesterol reduction. Propionate is the neoglycogen substrate for the liver, acting to increase adipogenesis and inhibit lipolysis in adipose tissues, which can neutralize cholesterol synthesis and lipogenesis in the liver. In addition, bacteria hydrolyze the urea that comes from the liver, forming ammonia and from it synthesize amino acids. They still synthesize vitamins, such as complex B and vitamin K [103]. 

A study evaluated the effects of diets rich in palmitic acid supplemented with DHA or ALA oil on the microbiota of rats [109]. They observed that the diet with an addition of 10% ALA by weight was responsible for an increase in the content of *Lactobacillus* and *Allobaculum*, which are species responsible for improving intestinal health and promoting the production of SCFA. These SCFAs increased their concentrations by 41.9% when compared to the group that received only palmitic acid [109].

Lecomte et al. [103] (Table 6) found that mice fed a HF diet (43% lipids) had a lower amount of Firmicutes and an increase in Bacteroidetes as compared to a group with a normolipid diet (12% lipids). This is correlated to the drastic decrease of *Lactobacillus* in the HF group, and appear to mainly decrease in obese phenotypes, as found in the experimental group of study. On the other hand, in Lam et al. [100] rats received one of two types of a diet, either a control (10% lipid energy) or a HF diet (60% energy derived from lipids where 24% was from SFA). The HF group showed an alteration in intestinal microbiota, with a decrease in Bacteroidetes strains and an increase in Firmicutes, as well as an increase in the inflammatory cytokines parameter. This finding was verified in an earlier study by Filippo et al. [110], in which they evaluated children who consumed two types of diets: one traditionally rural and one urban. In children consuming an urban diet, which included higher values of animal protein, starch, sugars, fats, and was poor in fiber, there was a predominance of Firmicutes and Preoteobacterias.

Another study [101] evaluated the effects HF diets supplemented with n-3 (EPA and DHA) or oleic acid would have on the metabolism of mice. The study consisted of two steps. In the first step, the mice were administered HF diets (60.3% of kcal from lipids) over an eight-week period. The second step consisted of a seven-week administration of these HF diets with the addition of either n-3 or oleic acid. As a result, they observed that the HF diet was responsible for increasing the concentration of Firmicutes and Enterobacteria, and decreasing the concentration of Bifidobacteria, but the second step did not present significant results. However, the n-3 group showed an increase of Firmicutes, while the group that received oleic acid decreased the concentration of Firmicutes as well as increasing the Bifidobacteria.

These microbial signals are responsible for regulating the release of Fasting Adipose Factor (Fiaf), which inhibits the action of lipoprotein lipase (LPL). The LPL hydrolyzes the triglycerides in a molecule of monoacylglycerol and two free fatty acids. When they enter the adipocyte, they are re-esterified and stored as fat, regulating this storage by Fiaf. SCFAs control the inflammatory response from a process in which they bind to the G protein conjugate receptors (GPCRs), thereby regulating the energy from the hormones that are derived from the gut [107].

Diets with n-6 PUFA are responsible for increasing the concentrations of Firmicutes, Actinobacteria, and Proteobacteria species and for decreasing the concentrations of Bifidobacteria [111]. Bifidobacteria are related to the increase in intestinal permeability that causes an increase in the circulation of LPS. LPS is associated with chronic systemic inflammation and metabolic syndrome, which includes the metabolic disorders of glucose and hypertriglyceridemia [111].

## 5. Fatty Acids and Non-Alcoholic Fatty Liver Disease

Non-alcoholic fatty liver disease (NAFLD) is another important disorder which contributes to obesity [112]. The exact NAFLD pathophysiology is unknown since it is a multi-factorial disease that encompasses one or more conditions which contribute to the metabolic syndrome, including diabetes mellitus, obesity, hypertension, and dyslipidemia [113]. NAFLD is considered a public health issue because it is one of the common chronic liver diseases in developed countries, found in, 20% to 30% of the population worldwide [114,115]. There are two pathological conditions with different prognoses: NAFLD is considered a condition without liver inflammation or hepatocytes damage, which may evolve into steatohepatitis with lobular inflammation and hepatocellular injury, called non-alcoholic steatohepatitis (NASH). One of the biggest problems caused by NASH is that many individuals with NASH may develop liver fibrosis. The latter may result in cirrhosis, hepatocyte death, and occasionally hepatocellular carcinoma, which involves a high likelihood of requiring a liver transplantation in the future [116].

Several therapeutic interventions, such as pharmacological and non-pharmacological, are proposed to treat NAFLD. Among the pharmacological therapies there are insulin sensitizers such as thiazolidinedione, lipid lowering drugs such as statins, antioxidants such as α-tocopherol, and vitamin D_3_ treatment. However, pharmacological approaches to treat liver steatosis are not always safe and effective [117,118].

Having an unhealthy lifestyle is an important factor influencing the development of NAFLD, mainly associated with a poor nutritional diet and physical inactivity. Therefore, non-pharmacological interventions have also been proposed as a strategy to reduce NAFLD severity. Among these non-pharmacological approaches are weight reduction, which involves strategies like bariatric surgery, some type of diets, and physical activity [119]. 

Nutritional approaches have been widely studied to reduce NAFLD severity. Dietary animal models and clinical trials in humans have been proposed to study new alternatives to reduce the risks and prevent NAFLD [120]. Although NAFLD pathophysiology is complex, it is strongly associated to oxidative stress, lipotoxicity, and inflammatory biomarkers in the liver. Plasma lipoproteins and fatty acid sources of liver triacylglycerol are derived from lipolysis in adipose tissue as nonesterified fatty acids. De novo *lipogenesis* (DNL) is a process that contributes to this lipotoxicity. During the fasting state, NAFLD patients display 26% of liver triacylglycerol derived from DNL, which is several times higher than the 5% observed in healthy individuals [121].

The quality of dietary fatty acids may have a role in the development of NAFLD, and conversely, may be an alternative source for decreasing deleterious NAFLD effects. Therefore, the composition of liver fatty acids may be involved in hepatic damage [122,123]. Dietary patterns are a combination of foods that are consumed by individuals and the amount of nutrients may produce synergistic health effects. The reason to study dietary patterns is because habitual food consumption is related to the human world diet [124]. 

The Mediterranean diet (MD) is a kind of dietary strategy that has been widely studied in metabolic dysfunction. According to Trichoppoulou, the MD has been defined as “primarily a plant-based diet characterized by a high ratio of monounsaturated fatty acids (MUFA) to SFAs with total fat accounting for 30–40% of daily energy consumption” [125]. In other words, MD is characterized by a high consumption of olive oil, as the main source of fat, vegetables, legumes, nuts, fruits, whole grains, fish, and seafood, with a low intake of meat and meat products, and moderate ethanol consumption, especially wine [125]. 

Recent studies have shown that the MD may have clinical nutritional effectiveness on the reduction of NAFLD [120,126] (Table 7). The ideal diet would result in a reduction of steatosis and an improvement in insulin sensitivity. A defect in insulin sensitivity is an important feature of NAFLD and DM II, which are two conditions that are closely related. In a randomized, cross-over six-week dietary intervention study, twelve non-diabetic subjects (six men and six women) with biopsy-proven NAFLD and at least three clinical features of metabolic syndrome (MetS), with the consumption of no more than seven to 10 standard alcoholic drinks per week, and without type 1 or 2 diabetes, were recruited to evaluate the effects of the MD on NAFLD and insulin resistance [126].

These patients used the MD and a control diet, which was a low-fat high-carbohydrate diet (LF/HCD), in random order with a six-week wash-out period in between. At the baseline, the subjects were obese with metabolic dysfunction parameters, such as elevated fasting concentrations of glucose, insulin, triglycerides, alanine aminotransferase (ALT), γ-glutamyl transpeptidase (GGT), and impaired insulin sensitivity. Weight loss was not observed between the two diets. Hepatic steatosis level after the MD was reduced in comparison to the LF/HCD and insulin sensitivity improved after the MD with a significant improvement in homeostatic model assessment for insulin resistance (HOMA-IR), but not in peripheral insulin resistance, measured by the glucose infusion rate (GINF) [126].

Gelli et al. demonstrated that the MD is associated with physical activity and may be considered as a safe therapeutic approach for reducing the severity of NAFLD. Forty-six adult patients were recruited, ranging from 26–71 years old with NAFLD within the previous six months of diet intervention. Although the MD approach was associated with physical activity, this correlation improved the steatosis grade in nine patients, and 25 out of 46 patients presented with weight reduction or maintenance. Moreover, several metabolic parameters, such as BMI, waist circumference, waist-to-hip ratio, ALT, GGT, serum glucose, total cholesterol/HDL, LDL/HDL, TG/HDL, HOMA, NAFLD score, and others showed a significant improvement between the baselines and the end of treatment [120].

Functional analyses of transcriptome data identified a group of genes from human NASH called Δ9 (stearoyl-coenzyme A desaturase 1 SCD-1), Δ5 (FADS1), and Δ6 (FADS2). Moreover, this study showed that hepatic fatty acid desaturation and unbalanced ω-6 to ω-3 ratio have an important role in the development of NASH. This study observed impaired desaturation fluxes in the ω-3 and ω-6 pathway, with augmented ω-6 to ω-3 ratio and a decreased ω-3 index, in fatty livers in both humans and mice (C57BL/6; six wild type fed with SCD and high fat diet (HFD)). Transgenic *fat-1* mice, which express a ω-3 desaturase, allowing the endogenous conversion of ω-6 into ω-3 fatty acids, were fed HFD [129]. 

Therefore, HFD-transgenic *fat-1* mice had a significant reduction in hepatic insulin resistance, were resistant to the adipogenic and steatogenic effects of HFD when compared to HFD-wild type mice, reduced macrophage infiltration, necroinflammation, and lipid peroxidation. They also reduced the expressions of genes involved in inflammation, fatty acid oxidation (fatty acid translocase—CD36/FAT and liver fatty acid binding protein L-FABP4), and lipogenesis (ACC, sterol response element-binding protein-1c-SREBP-1C, and fatty acid synthase—FASN). Afterward, they evaluated endogenous and exogenous ω-3 fatty acid enrichment on HFD-induced NASH, and these animals displayed similar findings as in the HFD-transgenic *fat-1* mice. In hepatocytes, CP24879, a Δ5/Δ6 desaturase inhibitor, significantly decreased intracellular lipid accumulation and inflammatory injury, and presented superior anti-inflammatory and antisteatotic actions in *fat-1* and ω-3-treated hepatocytes [129].

Some studies have evaluated the effects of PUFAs in adult individuals [102,127,128]. These human clinical trials demonstrated that PUFA supplementation, especially fish oil, may be an important alternative dietary therapy on NASH. Seventy-eight patients diagnosed with NASH were enrolled and randomly assigned into either the control group or the PUFA treated group (50 mL of PUFA with 1:1 DHA: EPA added to the daily diet) for six months. The group observed that after six months of treatment, these patients displayed a considerable improvement in several NASH parameters, including ALT and AST levels, triacylglycerol (TG), total cholesterol (TC) levels, systemic inflammatory markers, such as C-reactive protein (PCR) and malondialdehyde (MDA), and fibrosis parameters, like type IV collagen and pro-collagen type III pro-peptide, were also significantly reduced after treatment [127]. 

Similar results were seen in a randomized clinical trial that aimed to assess the effects of fish oil on NAFLD and hyperlipidemic patients. Eighty individuals with NAFLD and hyperlipidemia were randomly assigned to consume either two capsules of fish oil twice per day, including 182 mg EPA and 129 mg DHA, or two capsules of corn oil twice per day, without EPA and DHA, but containing vitamin E, gelatin, glycerin, and water. In addition to vitamin E, gelatin, glycerin, and water, the total capsule weight was 1000 mg. The capsules were taken for three months in a double-blind, randomized clinical trial. This study found a high plasma concentration of EPA and DHA in the fish oil group after intervention and a significant reduction in TG, TC, apolipoprotein B, glucose, ALT, and GGT, and significantly increased serum adiponectin levels. Some NAFLD biomarkers, such as fibroblast factor growth 21 (FGF-21) and CK18 fragment M30 (CK18-M30), and pro-inflammatory cytokines, tumor necrosis factor-α (TNF-α), leukotrienes 4, and prostaglandin E2, decreased after fish oil intervention in NAFLD/dyslipidemic patients. Corn oil increased creatinine serum levels, but had no other metabolic effects [102]. 

Hodson et al. performed a randomized sub-study with 16 NAFLD participants that received four g/day EPA with DHA, while another group consumed a placebo for 15–18 months. Individuals with NAFLD, who had an increase in the erythrocyte DHA enrichment of ≥2% with the treatment of ω-3 FA, showed positive changes in hepatic insulin sensitivity and hepatic lipid metabolism. Erythrocyte DHA enrichment is a kind of surrogate marker of changes in tissue enrichment and may be associated with alterations in hepatic DNL, postprandial FA partitioning, and hepatic and peripheral insulin sensitivity. The results demonstrated that although erythrocyte DHA enrichment ≥2% had no effect in diminishing fat liver content, this fat liver reduction may be due to the decrease in hepatic DNL with concomitant increase in hepatic FA oxidation and hepatic insulin sensitivity. This reduction in fat liver was associated with improved hepatic insulin sensitivity, but was not related to peripheral insulin sensitivity [128].

In animal models, several studies have observed beneficial effects of PUFAs on NAFLD. NAFLD may be induced through a HFD diet intervention in mice and rats (Table 8). Wang et al. showed that C57BL/6 mice fed with a HFD for four days induced lipid accumulation, however, short-term n-3 PUFA-enriched HFD (ω-3HFD) reversed this effect. A metabolomics assay was able to determine the reduced plasma content of hydroyeicosapentaenoic acid (HEPEs) and the epoxyeicosatetraenoic acid (EEQ) in short term-HFD animals and, after ω-3 supplementation, these FAs increased. Furthermore, ω-3HFD was able to reduce the macrophage infiltration in adipose tissue and pro-inflammatory cytokines (IL-6, MCP-1, and TNF-α) in the plasma. Primary hepatocytes and peritoneal macrophages were used to evaluate the mechanisms. Therefore, the activation of pro-inflammatory cytokines, as well as the activation of the JNK pathway by palmitate in macrophages, decreased with a mixture of 17,18-EEQ, 5-HEPE, and 9-HEPE, which are identified as the efficient components of these metabolites, including HEPEs and EEQs. Herein, the results have demonstrated that the mixture (17,18-EEQ, 5-HEPE, and 9-HEPE) may be an alternative therapy to prevent the early stages of NAFLD by inhibiting adipose tissue macrophage infiltration and systemic inflammation via cJun-N-terminal-kinase (JNK) signaling [123].

Positive effects of a DHA/EPA-enriched diet on NAFLD after eight or 12 weeks was observed in another study, as the quality of dietary lipids modulated some gene expressions. A liver transcriptoma is an analysis used to evaluate many hepatic processes like transcription (histone methylation/acetylation, chromatin modification), translation (mRNA, rRNA, and tRNA), protein turnover (polyubiquitination), and protein transport, metabolism of lipids and fatty acids, lipid/sterol metabolism, lipid/fatty acid biosynthesis, lipoprotein transport, and cholesterol/phospholipid efflux. After transcriptoma analysis, we concluded that the quality of dietary fat could modulate PPAR-related gene expression, since corn-oil based HFD induced PPAR-γ gene signatures, while DHA/EPA-enriched diets induced genes known to be regulated by PPAR-α [130].

In addition to these positive effects, Bargut et al. investigated if a diet rich in fish oil (HFO n-3 PUFA) for eight weeks could have hepatic alterations in HFD-induced NAFLD. The group that was fed with HFD displayed obesity, liver damage, hypertriglyceridemia, hepatic insulin resistance, and steatosis accompanied with an increase in hepatic lipogenesis and a decrease in beta oxidation. However, the HFO group did not present with metabolic alterations like the HFD group, with improvement in hepatic glucose output with reduced expression of genes related to lipogenesis via SREBP-1C and FAS improved inflammatory markers, with an increase in adiponectin levels as well as elevated beta oxidation with increased expression of PPARα and the PPAR-α target gene, Carnitine palmitoyltransferase I (CPT-1), which is considered the master regulator of mitochondrial beta oxidation [131]. 

A current study evaluated the ideal ratio of DHA/EPA supplementation in HFD-liver damaged mice. Shang et al. assessed different ratios (1:2, 1:1, and 2:1) of DHA/EPA supplementation for 11 weeks. DHA/EPA supplemented mice displayed a reduction in several parameters, and the best DHA/EPA ratio was found to be 1:2. The results indicated that the DHA/EPA ratio of 1:2 could increase HDL/C levels when compared to the other ratios, with a greater reduction in ALT, AST, and MDA levels, and increased glutathione (GSH) levels. It also reduced the expression of lipid metabolism genes, such as Sterol regulatory element-binding protein-1-C (SREPB-1C), Stearoyl-CoA desaturase-1 (SCD-1), Acetyl-CoA carboxylase (ACC-1), and PPAR-γ, lowered the expression of proteins c-Jun and c-Fos levels, which are proteins related to inflammatory responses of metaflammation, activating protein-1 (Ap-1), and weakening the activation of Ap-1. Additionally, serum levels of pro-inflammatory cytokines (IL-6 and IL-1β) were reduced with the DHA/EPA ratio of 1:2 [132]. 

Another animal model that demonstrated steatohepatitis is Ldlr^−/−^ mice, which is a Western diet (WD)-induced hepatic fibrosis animal model. This model provides considerable insight into the similarity of processes that are related to cardiovascular diseases and the development of NASH, but are not identical to the process in humans [133]. Some studies focused on evaluating the effects of WD to induce NASH in Ldlr^−/−^ mice [134,143]. Mice were fed with WD supplemented with olive oil (OO) (WD + OO), EPA (WD + EPA), DHA (WD + DHA), and DHA + EPA (WD + DHA/EPA) for 16 weeks. Ldlr^−/−^ mice that were fed with WD + OO displayed a severe NASH phenotype, accompanied with inflammation, oxidative stress, and fibrosis. The results demonstrated that both DHA and EPA were able to decrease ALT and AST in WD + OO groups. However, considering the other parameters that characterize the severity of NASH, WD + DHA could reduce the expression of most of these parameters, such as cell surface markers for Kupffer cells and macrophages in the liver (C-type lectin domain family 4f—Clec4f; C-type lectin domain family 10a—Clec10a; cell determination-68—CD68; and F4/80) when compared to the other groups [133]. 

Furthermore, MD+DHA have diminished inflammatory markers, such as IL-1β, TNF-α, toll-like receptor-4 (TLR4), and -9 (TLR-9). MD+DHA also had genes involved in the TLR pathway cluster of differentiation-14 (Cd-14) and myeloid differentiation in the primary response gene-88 (MyD88) and had blocked WD-induced accumulation of nuclear factor κ beta (NFκB) in hepatic nuclei. Dietary DHA was more able to reduce oxidative stress (NADPH oxidase subunits Nox2, p22phox, p40phox, p47phox, and p67phox) as compared to EPA, and had diminished procollagen-1a1 (Procol1α1), a marker of stellate cell marker, and had decreased cytokine TGF-β1, which is a cytokine involved in the activation of hepatic stellate cells and Procol1α1 [133].

The effectiveness of DHA in WD-induced NASH Ladlr^−/−^ mice was compared to EPA by a metabolomics analysis that focused on changes in hepatic lipid, amino acid, and vitamin metabolism. In NASH, hepatic sphyngomielin, SFA, MUFA, and n-6 PUFA accumulate, with a depletion in n-3 PUFA. Hence, dietary n3-PUFAs has the ability to reduce hepatic sphyngomielin, SFA, MUFA, and n-6 PUFA and also decrease the hepatic nuclear abundance of NFκB in NASH-linked inflammation [133]. 

Hepatic fibrosis involves a significant production of extracellular matrix (ECM), from activated hepatic stellate cells, and myofibroblasts that infiltrate the liver. Several subtypes of collagens underlie the connective tissue in the liver; therefore, fibrosis, which is the result of hepatic damage, is connected to an increase ECM deposition of collagen type 1 (collagen 1 A1-Col1A1) and also is associated with a high level of production of proteins from stellate cells and macrophages that are involved in ECM remodeling. Thus, another explanation as to how DHA and EPA differentially affect WD-induced hepatic fibroses is associated to the TGF-β pathway. WD+DHA did not increase the hepatic nuclear abundance of phospho-mothers against decapentaplegic homolog (Smad3) when compared to WD+OO and WD+EPA, which increased Smad3 expression. Smad3 is a key regulator of Col1A1 expression in stellate cells. Human LX2 stellate cells were treated with DHA and there was a blocked TGF-β mediated induction of Col1A1, concluding that DHA decreased the WD-induced fibrosis through the TGF-β-Smad3-Col1A1 pathway [134].

In rats, current studies have evaluated dietary fatty acids on NAFLD. Sprague-Dawley rats were fed with HFD and supplemented with different oils for 12 weeks, divided in different groups: (i) high oleic canola oil (HOC); (ii) conventional canola oil (C); (iii) conventional canola oil/flax oil blend (C/F) (3:1 ratio); (iv) high linoleic safflower oil (SF); (v) soybean oil (SB); (vi) lard and soybean oil (L); and, (vii) a weight-matched group fed lard and soybean oil (WM). The results demonstrated that the C/F group had decreased hepatic steatosis, presented the lowest concentration of fat liver, as did the WM group, and had an altered hepatic phospholipids fatty acid profile by increasing EPA and DHA. All of the groups that contained canola oil (HOC, C, and C/F) gained the least amount of body weight during the study, and after 12 weeks of diet, these groups displayed the lowest weight gain without differences in adiposity, which was assessed by visceral fat mass. The C/F diet contained MUFA and high amounts of alpha-linolenic acid (ALA), a plant-based n-3 PUFA, which was demonstrated to be beneficial for diminishing hepatic steatosis in HFD-Sprague-Dawley rats [135].

One example of a plant that is rich in ALA is *Perilla frutenses*, which is a medicinal plant that is found in East Asia and India, and the oil from the seeds oil contain 60% ALA. Chen et al. investigated the role of perilla oil in high-fat /high-cholesterol diet (HFD/HC), inducing NASH. Two groups of Sprague-Dawley rats were fed either HFD/HC or fed perilla oil-enrichment HFD (POH) for 16 weeks. The results demonstrated that the POH group showed improvement in HFD-induced hyperlipidemia (TG, CT, and LDL), reduced hepatic steatosis with reduced ALT activity, reduced AST enzymes, reduced hepatic inflammatory infiltration around the portal area, and reduced HFD-induced hepatic fibrosis. On the other hand, perilla oil could not modulate the expression of genes that are involved in cholesterol synthesis, but increased cholesterol removed hepatocytes by conversion to bile acids and increased fecal cholesterol excretion. HFD downregulated ABC proteins, including ATP-binding cassette hemitransporters G5 and G8 (ABCG 5 and ABCG 8), which are involved in cholesterol secretion, so these effects were pronounced in the POH group. Moreover, perilla oil increased the expression of Cytochrome P-450 2E1 (CYP2A1) and CYP27A1, which are two key enzymes in bile acid production, whereas the HFD/HC group had reduced the expression of these enzymes [136].

Despite many studies presenting several beneficial effects of n-3 and perilla oil, which contains a large amount of ALA, on NASH, a few studies have reported no benefits after consuming n3-PUFAs on NASH [137,138]. Du et al. demonstrated that EPA supplementation accentuated hepatic triglyceride accumulation in mice with impaired fatty acid oxidation. C57BL/6 mice were fed with HFD, either supplemented or not with 3% EPA, in the presence or absence of 500 mg mildronate/kg/day for 10 days. Milindronate decreases hepatic carnitine concentration and mitochondrial FA β-oxidation. After dietary EPA supplementation, mildronate-induced triglyceride accumulation was exacerbated, with considerable increase in EPA and a decrease in the total n-3/n-6 ratio. Conversely, EPA supplementation decreased the mildronate-induced mRNA expression of inflammatory genes, such as macrophage-expressed gene 1 (MPEG1), cyclooxygenase 2 (COX 2), CD68, F4/80, and increased G protein–coupled receptor 120 (GRP120), a protein related to mediate the anti-inflammatory effects of n-3 PUFA, in adipose tissue [137]. 

Provenzano et al. observed that Balb/C mice fed a methionine and choline deficient (MCD) diet, an animal model of steatohepatitis, for four or eight weeks. Along with the diet, the animals were either supplemented n-3 PUFA and n-9 MUFA (EPA/DHA 25 mg with OO 75 mg) (MCD/n-3) or supplemented with n-9 MUFA alone (OO 100 mg) (MCD/OO) two times per week by intragastric gavage. After eight weeks, the MCD/n-3 group displayed higher levels of ALT, severe scores for inflammation, increased intrahepatic expression of inflammatory markers, such as TNF-α and C-C motif chemokine ligand 2 (CCL2), increased expression of profibrogenic genes TGF-β1, and tissue inhibition of metalloproteinase (TIMP-1) with higher portal pressure as compared to MCD/OO. Moreover, after hepatic fatty acid profile analysis, t supplementation was confirmed to result in effective n-3 incorporation. The results showed that the addition of specific nutrients may modulate the course or the progress of steatohepatitis, indicating further attention and monitoring is required when administering n-3 PUFA in patients with hepatic inflammation [138].

A current study showed that extra virgin olive oil (EVOO) displayed a protective effect on the inflammatory response and liver damage in a NAFLD-mouse model. C57BL/6 mice were fed with standard chow diet (SCD) and HFD based on lard, where 49% of the energy was from fat, for 12 weeks to NAFLD development. The mice that were fed with HFD were divided into four groups: (i) unchanged HFD-L (HFD-L); (ii) HFD based on EVOO (HFD-EVOO); (iii) HFD based on EVOO rich in phenols (HFD-OL with the same percentage of fat); and, iv) R (reversion, LFD) over a period of 24 weeks. EVOO diets were able to reduce body weight and improve the plasma lipid profile, the pro-inflammatory cytokines in the epidydimal adipose tissue, such as IFN-γ, IL-6, and leptin, and improve the macrophage infiltration [139]. 

Moreover, EVOO decreased the NAFLD activity (NAS) score and increased the hepatic adiponutrin (Pnpal3), which is a protein that plays a role in triglyceride metabolism by acting as a hydrolase. Also, Cd36 gene expression, which is a gene responsible for fatty acid uptake, esterification into triglycerides, and contributes to fatty liver in HFD-fed mice, was increased in the EVOO groups. Hepatic fat composition showed an increase in MUFAs, especially oleic acid, and a decreased amount of SFAs. In conclusion, the results suggested that methionine metabolism, which influences DNA methylation status may induce the modifications in the expression of selected genes that are central to lipid metabolism in HFD-EVOO mice and to the cell cycle in HFD-OL mice [139].

Palmitoleate is a MUFA (16:1 n7) and is available as a dietary source and is produced by adipose tissue. It is a bioactive lipid and may coordinate metabolic crosstalk between the liver and adipose tissue [144]. Mice were fed with a low-fat diet (LFD) for 12 weeks. One group was supplemented with palmitoleate and the control group with oleate for a period of four weeks. Palmitoleate was able to improve systemic insulin-sensitivity, induce hepatic steatosis, but improve insulin signaling in the liver with a significant increase in insulin-stimulate Akt (Ser 473) phosphorylation. Furthermore, palmitoleate reduced phosphorylation of NFκB p65 (Ser468), IL-6, and TNF-α. In hepatocytes, palmitoleate increased fat deposition, stimulated FAS expression, activated SREBP-1c, and decreased inflammation (NFκB p65 Ser 68, TNF-α, and IL-6) in both hepatocytes and RAW macrophages. Despite palmitoleate inducing hepatic steatosis, this FA may dissociate the liver inflammatory response from hepatic steatosis, and promote insulin-sensitization and its pro-lipogenic effect, by enhancing hepatic FAS expression due to higher expression of SREBP-1c [140]. 

Conversely, the excess consumption of saturated fatty acids (SFAs) may be a risk factor for NAFLD pathogenesis [121]. Palmitic acid (PA), which is a kind of SFA, in cooperation with receptor toll-like type 2 (TLR2) have been shown in vitro to activate inflammation in the development of NASH. Kupffer cells and hepatic stellate cells (HSC) were isolated from wild type mice and stimulated with TLR2 and palmitic acid. These cells responded to the TLR2 ligand, but when they were stimulated with PA alone, increased TLR2 signaling-targeting genes were not seen, including cytokines and inflammasome components. Kupffer cells were more important than HSC in the TLR2-mediated progression of NASH, since the TLR2 ligand could increase the Nod-like receptor protein 3 (NOD3), which is an inflammasome component in Kuppfer cells. Moreover, PA together with the TLR2 ligand have induced caspase-1 activation and the release of interleukin-1β (IL-1β) and -1α (IL-1α) in Kuppfer cells [141]. 

Toll-like receptors are a defense of the organism against invading pathogens by proinflammatory cytokines in immune cells, but when TLR signaling is overactivated, altering the TLR tolerance, these conditions may result in a large number of proinflammatory cytokines that lead to tissue damage [145]. On the other hand, inflammasome activation is a pathway that converts pro-interleukin-1β into secreted IL-1β and may be induced by endogenous and exogenous danger signals. Lipopolyssacharide (LPS), a toll-like receptor 4 (TLR4) ligand, activates inflammasome and plays a role in NASH. Other studies have demonstrated that PA has activated inflammasome and induced sensitization in the LPS-induced-IL-1β release in hepatocytes, releasing danger signals from hepatocytes in a caspase-dependent manner. Thus, hepatocytes may orchestrate tissue responses to danger signals in NASH [146].

Another study evaluated the role of peroxidized oil in steatohepatitis and hepatic inflammation. Corn oil (CO), in which linoleic acid is the main FA, contains peroxidized FAs. Han-Wister rats were treated with CO (PO), unperoxidized FA (OIL), or tap water (WA), and applied by gavage over a period of six days. The PO group displayed a pro-oxidant state with enhanced NO-synthetase-2 (NOS-2), NO-formation, pronounced lipid peroxidation, and a decrease in α- and γ-tocopherol in the liver. Furthermore, the PO group had an increase in inflammatory markers, such as TNFα, COX-2, and IL-1β, and macrophage markers cd68 and cd 163 in the liver. In hepatocytes, endothelial and Kupffer cells were isolated from the untreated liver and incubated with peroxidized linoleic acid; the linoleic acid increased the secretion of TNF-α, mRNA expression of TNF-α, NOS-2, COX-2, and p38MAPK phosphorylation expression, especially in Kupffer cells. When p38MAPK was inhibited, an increase in NOS-2 and COX-2 mRNA in linoleic acid-induced Kupffer cells was seen, indicating that p38MAPK activation may be involved in the pro-inflammatory effects of linoleic acid [142].

## 6. Conclusions

This review evaluated the consumption of saturated and unsaturated fatty acid sources, including MUFAs or PUFAs (EPA and DHA), during in vivo, in vitro, and in human studies. PUFAs may promote benefits for obesity-related comorbidities, such as a reduction in insulin resistance, dyslipidemias, inflammation, and non-alcoholic fatty liver disease markers. The HF diets, with a predominance of saturated fatty acids, influenced intestinal permeability damages, leading to the greater stimulus of endotoxin production and consequently greater inflammatory process. However, due to the different types of SFA sources, this lipid class deserves further study, especially on the dyslipidemia profile. On the other hand, ingesting higher concentrations (1000 mg/day) of EPA and DHA may be a great supplementation option, together with a dietary fatty acid balance, which may promote the prevention and decrease of the metabolic framework of obesity and its disorders. 

## Figures and Tables

**Table 1:** Effects of different types of fatty acids on insulin resistance and associated comorbidities during human studies.

Host	Fatty Acid Composition	Glycaemia-Related Effects in Obesity	References
Humans	Diets with 63% SFA (42% palmitic, 29% MUFA, 4% PUFA)	Increased glycaemia (3.70%) Increased insulin (25%)	[22]
Hypertensive women with DM II	(1) 1.5 g fish oil (21.9% EPA, 14.1% DHA) (2) 2.5 g fish oil (21.9% EPA, 14.1% DHA) (3) Control group.	Glucose, mg/dL; glycated hemoglobin, %; insulin, µU/mL and HOMA-IR without changes.	[26]
Diabetics and nondiabetics individuals	(1) 300 g of vegetables and 25 mL of PUFA-rich plants (61.8% linoleic, 11.5% linolenic, and 16.4% of oleic fatty acid) per day	Reduction of HbA1c (hemoglobin A1c) (%) after 4 and 8 weeks	[27]
Subjects with early-stage DM II or metabolic syndrome	Individuals received corn oil (CO); a combination of borage [*Borago officinalis* L.] and echium oil [*Echium plantagineum* L.] (BO) or fish oil (FO): 9 CO capsules, 10 BO capsules (3 borage and 7 echium), or 9 FO capsules	Statistically significant increase in insulin and reduction in HbA1c of FO group.	[28]
DM II subjects	Supplementation of 3 g/day of ALA or placebo for 60 days	ALA group improved IS corrected for FFM (M/FFM)—Insulin sensitivity corrected for fat-free mass.	[29]
DM II subjects	(1) High-carbohydrate/high-fiber/low-glycemic index diet (CHO/fiber group) (2) High-MUFA diet (MUFA group) (3) High-carbohydrate/high-fiber/low-glycemic index diet plus physical activity program (CHO/fiber + Exercise group) (4) High-MUFA diet plus physical activity program (MUFA + Ex group).	Reduction of HbA1c levels in the MUFA group.	[30]
Human clinical trials: obese children	Supplementation of CLA (3 g/day) with 50:50 isomers c9, t11, and t10, c12 or placebo (1 g/day) 3 times per day for 16 weeks	Significant improvement in insulin, fasting insulinemia, and HOMA-IR in CLA group.	[31]

Abbreviations: Saturated fatty acids (SFA); monounsaturated fatty acids (MUFA); polyunsaturated fatty acids (PUFA); type II diabetes mellitus (DM II); docosahexaenoic (DHA) fatty acids; alfa-linolenic fatty acid (ALA); homeostasis model assessment-estimated insulin resistance (HOMA-IR); hemoglobin A1c (HbA1c); insulin sensitivity corrected for fat-free mass (FFM); carbohydrate (CHO); conjugated linoleic acid (CLA).

**Table 2:** Effects of different types of fatty acids on insulin resistance and associated comorbidities during in vivo and in vitro studies.

Host	Fatty Acid Composition	Glycaemia-Related Effects in Obesity	References
In vitro insulin resistance at cellular level from thoracic aorta arteries of three 8-week-old wild-type male mice	Cell lines were cultured with high glucose and were serum-starved for insulin signaling and relatives free fatty acids (palmitate or oleate)	Oleate treatment for 2 h did not produce insulin resistance. Palmitate significantly induced insulin resistance for 18 h.	[36]
C57BL/6 male mice	SFA High Fat Diet (HFD) with 45% palmitic acid; MUFA-HFD (45% oleic acid), and a standard chow as a control group (5.2% fat: 0.9% SFA, 1.3% MUFA, and 3.4% PUFA)	Lower fast glucose, insulin concentrations and insulin secretion in MUFA-HFD group compared to the SFA-HFD group.	[40]
Hypertriglyceridemia-induced dyslipidemia rats	High sucrose diet supplemented with either sunflower oil or Conjugated Linoleic Acid (CLA) (2 g/100 g diet)	Decrease in glucose and insulin (mmol/L) in CLA supplemented group.	[39]
Diet-induced IR rat model	Supplementation of fish oil (n-3 PUFA), sunflower oil (n-6 PUFA), and high oleic sunflower oil (n-9 MUFA)	Reduction of HOMA-IR in n-3 PUFA.	[43]

Abbreviations: Saturated fatty acids (SFA); monounsaturated fatty acids (MUFA); polyunsaturated fatty acids (PUFA); homeostasis model assessment-estimated insulin resistance (HOMA-IR); conjugated linoleic acid (CLA); high fat diet (HFD).

**Table 3:** Effects of consuming different types of fatty acids during human studies on dyslipidemia.

Host	Fatty Acid Composition	Effects	References
Humans with hypertriglyceridemia	n-3 PUFA (2,3 and 4 g of fish oil)	Reduction in VLDL, TG, non-HDL, LDL and Apo-B	[62]
Humans: Hemodialysis Patients	2 capsules of EPA and 1.28 g DHA/day	TG, TC, and LDL (no differences) EPA/DHA and placebo. Increase in HDL.	[65]
Humans	2 capsules of 900 mg/day containing EPA and DHA	Increase in HDL, reduction in LDL and TG. Improvement Protein C reactive levels.	[66]
Humans	4 capsules of 1 g/day containing EPA and DHA for 6 months	Reduction in TG, increase in HDL. No difference in TC and LDL.	[67]
Humans	4 different foods enriched with 3 rich-n-3-PUFA oils	Increase in HDL. LDL—no differences.	[68]

Abbreviations: Polyunsaturated fatty acids (PUFA); very low density lipoprotein (VLDL); triacylglycerol (TG); unlike LDL-C (non-HDL); low density lipoprotein (LDL); apolipoprotein-B (Apo-B); total cholesterol (TC); eicosapentaenoic (EPA); docosahexaenoic (DHA); high density lipoprotein (HDL).

**Table 4:** Effects of consuming different types of fatty acids during in vivo studies on dyslipidemia.

Host	Fatty Acid Composition	Effects	References
Wistar rats	Three diets and a control group (7% fat): CG (Saturated fatty acid); SO (Sesame oil—oleic and linoleic fatty acid); FO (Flaxseed oil—alfa-linolenic fatty acid), and SFO (flaxseed and sesame oil)	Increased levels of total cholesterol, HDL, VLDL, and TAG in CG and SO groups. Reduction in levels in non-HDL and LDL for SFO group.	[76]
Wistar rats	6 groups: control (AIN-93G—7% soy oil); extra virgin oil (OO-C) (7% soy oil and 13% extra virgin); sunflower oil (HOSO) (7% soy oil and 13% sunflower oil); Atherogenic diet (AT), (rich-SFAs (12.3 g %) and cholesterol (4 g %); Experimental diets were: OO and HOSO (11.82% and 12.9 g % MUFA and 4% cholesterol).	HOSO: Increase in TC and non-HDL, HDL diminished and decrease in TG in comparison to AT. OO: Reduced TC and non-HDL.	[79]
Wistar rats	4 groups over 5 weeks: Extra virgin olive oil group (OO) (SFA 12.0%, MUFA 81.9%, PUFA 6.10%), sunflower group (HOSO) (SFA 7.82%, MUFA 87.11%, PUFA 4.75%), sunflower oil and phytosterols group (HOSO-F) (1% phytosterols); sunflower oil and n-3-PUFA (HOSO-P) (6.5% fish oil).	HOSO: Increase in TC and non-HDL and reduction in HDL; HOSO-P and HOSO-F: Decrease in TC, non-HDL and TAG and increase in HDL in comparison to the OO group.	[80]
Wistar rats	High fat (HF) diets enriched in saturated fatty acids (SFAs); MUFA (oleic acid); PUFA n-6 and PUFA n-3.	TG decreased in MUFA and PUFA n-6 just at first day; Reduction in TG levels with a longer time feeding (21 days)	[81]

Abbreviations: Polyunsaturated fatty acids (PUFA); very low density lipoprotein (VLDL); triacylglycerol (TG); unlike LDL-C (non-HDL); low density lipoprotein (LDL); total cholesterol (TC); high density lipoprotein (HDL); American Institute of Nutrition Rodent Diets for growth (AIN-93G); sunflower group (HOSO); Extra virgin olive oil group (OO);

**Table 5:** Effects of different types of fatty acids on the inflammatory process and intestinal microbiota in human studies.

Host	Fatty Acid Composition of the Experiment	Microbiota	Inflammatory Process	References
Adults individuals	Control group (28.4% fat, of which 5.3% was palmitic fatty acid and 15.9% was oleic fatty acid); High fat (40.4% fat, of which 16% was palmitic fatty acid and 16.2% was oleic fatty acid); High fat (40.4% fat, of which 2.4% was palmitic fatty acid and 28.8% was oleic fatty acid)	Not observed	↓ IL-1β, IL-10, IL-18, and TNF-α ↑ IL-1β, IL-10, IL-18, and TNF-α	[93]
Obese children and adolescents (BMI >95th percentile for sex and age)	Therapeutic protocol: ↓ Fat ↓ Sugar ↑ Fibers	Not observed	↓ IFN-γ, IL-12A, IL-18, TNF-α, IL-6, IL-1β.	[90]
Adult individuals	Control group (20% fat/olive oil—MUFA) High fat with n-3 PUFA (35% fat with fish oil) High fat with n-6 PUFA (35% fat and grapeseed oil) High Fat with SFA (35% fat and coconut oil)	Not observed	↓ endotoxins postprandial ↑ endotoxins postprandial	[91]
Obese individuals	Mediterranean Diet (35% fat, 22% monounsaturated) Low-fat, high-complex carbohydrate diet diet (28% fat, 12% monounsaturated)	↑ *Roseburia* and *Oscillospita* and ↓ *Prevotella* ↑ *Prevotella*, ↓ *Roseburia* and ↑ *F. prausnitzii*	Not obeserved	[94]
Metabolic syndrome “at-risk” population	HS: High saturated fatty acids diet High monounsaturated fat (MUFA)/high glycemic index (GI) (HM/HGI) High MUFA/low GI (HM/LGI) High carbohydrate (CHO)/high GI (HC/HGI) High CHO/low GI (HC/LGI)	↑ Bifidobacterium and Bacteroidetes		[95]
Hypercholesterolemic individuals	Virgin olive oil (OO) naturally containing 80 mg of PC/kg, (VOO) Phenolic compound (PC) enriched virgin olive oil containing 500 mg PC/kg, from OO (FVOO) PC-enriched virgin olive oil containing a mixture of 500 mg PC/kg from OO and thyme 1:1 (FVOOT)	↑ Bifidobacterium, Parascardovia denticolens and Roseburia		[96]
DM 2 subjects	Control group Sardine group (SG)	↓ Firmicutes/Bacteroidetes ↓ Firmicutes/Bacteroidetes and ↓ bacteroidetes/prevotella	↑ TNF-α ↑ Adiponectin	[97]

Abbreviation: Interferons-γ (IFN-γ); body mass index (BMI).

**Table 6:** Effects of different types of fatty acids on the inflammatory process and intestinal microbiota in in vivo studies.

Host	Fatty Acid Composition of the Experiment	Microbiota	Inflammatory Process	References
Female rats	Control group (10% kcal fat), high Fat (60% kcal fat, of which 34% was SFA)	↑ Firmicutes and ↓ Bacteroidetes	↑ Inflammatory citokines	[100]
Female mice	Control group (12.6% fat) High fat (60.3% fat) High fat with oleic fatty acid High Fat with n-3 PUFA (EPA and DHA)	↑ Firmicutes and Enterobacteria, ↓ Bifidobacteria ↓ Firmicutes and ↑ Bifidobacteria ↑ Firmicutes	Not observed	[101]
Male rats	Control group with palmitic fatty acid Palmitic fatty acid with DHA Palmitic fatty acid with ALA	↑ Lactobacillus ↑ Lactobacillus and Allobaculum, ↓ Proteobacteria	Not observed	[102]
Elderly male rats	Normolipid diet (12% fat) High Fat (43% fat)	↓ Firmicutes ↓ Lactobacillus	Not observed	[103]
Male rats	Placebo (10% skimmed milk) High Fat with placebo Placebo with 1 × 109 CFU. *B. pseudocatenalatum* High Fat diet with 1 × 109 CFU. *B*. *pseudocatenalatum*	↑ Firmicutes (65%) and Bacteroidetes (31%) ↑ Firmicutes, ↓ Bacteroidetes, ↑ Proteobacteria ↑ Firmicutes (66%) and Bacteroidetes (31%) ↑ Firmicutes, ↓ Bacteroidetes	↑ CD8^+^/CD4^+^, ↑ TNF-α, MCP-1, IL-10, IL-17A, IP-10, IL-6, ↑ LPS ↓ CD8^+^/CD4^+^, ↓ TNF-α, MCP-1, IP-10, 1L-17A, IL-6, ↓ LPS	[90]
Male rats	Normolipid diet (10% fat) with Nopal (4% fiber) High fat (46% fat) with Nopal (4% fiber)	↑ Firmicutes ↑ Bacteroidetes	↑ IL-6 ↓ IL-6, ↓ in adipocyte size	[99]
Male rats	Control group Control group with high sugar (HS) High fat High fat with HS	Not observed	↑ size of adipocytes and hepatocytes ↑ TNF-α ↑ IL-6, ↑ IL-1 β	[98]

Abbreviations: Saturated fatty acids (SFA); docosahexaenoic (DHA) fatty acids; eicosanoic acid (EPA); colony-forming unit (CFU); CD4 and CD8 T cell surface molecules; tumor necrosis factor alpha (TNF-α); monocyte chemoattractant protein-1 (MCP-1); interleukin(IL); interferon induced protein (IP); lipopolyssacharide (LPS).

**Table 7:** The effects of dietary fatty acids in humans with non-alcoholic fatty liver disease (NAFLD).

Host	Fatty Acid Composition	Effects	References
Human Clinical Trial: Adults	- Mediterranean diet: olive oil, vegetables, legumes, nuts, fruits, whole grains, fish and seafood, moderate wine - Low-fat-high carbohydrate diet (LF/HCD) Duration: 6 weeks (6-week wash-out period in-between)	- Weight loss was not observed between the two diets - Reduced hepatic steatosis - Improved insulin sensitivity (HOMA-IR) - No differences in peripheral insulin resistance	[126]
Human Clinical Trial: Adults	- Mediterranean diet and Physical activity Duration: 6 months	- Improved BMI, waist circumference, waist-to-rip ratio, ALT, GGT, serum glucose, total cholesterol/HDL, LDL/HDL, TG/HDL, HOMA, NAFLD score	[120]
Human Clinical Trials: Adults	n-3 PUFAs - (50 mL of PUFA with 1:1-DHA: EPA into daily diet) Duration: 6 months	- Reduced ALT and AST levels - Reduced triacylglycerol (TG), total cholesterol (TC) levels - Reduced systemic inflammatory markers: C-reactive protein (PCR) - Reduced pro-oxidant factors: malondialdehyde (MDA) - Reduced fibrosis parameters: type IV collagen and pro-collagen type III pro-peptide	[127]
Human Clinical Trials: Adults	n-3 PUFAs - 2 capsules fish oil 2 times per day (182 mg EPA and 129 mg DHA) - 2 capsules corn oil 2 times per day (without EPA and DHA) Duration: 3 months	- Reduced TG, TC, apolipoprotein B, glucose, ALT, GGT. - Increased serum adiponectin levels. - Reduced NAFLD biomarkers: fibroblast factor growth 21 (FGF-21) and CK18 fragment M30 (CK18-M30). - Reduced pro-inflammatory cytokines: tumor necrosis factor-α (TNF-α), leukotrienes 4, and prostaglandin E2. - Corn oil increased creatinine serum levels, but without other metabolic effects.	[102]
Human Clinical Trials: Adults	n3-PUFAs 4 g/day EPA and DHA - Placebo Duration: 15–18 months	- Erythrocyte DHA enrichment ≥2%: no changes in fat liver content. - Fat liver reduction: decrease in hepatic DNL with concomitant increase hepatic FA oxidation and hepatic insulin sensitivity.	[128]

Abbreviations: alanine aminotransferase (ALT); γ-glutamyl transpeptidase (GGT); triacylglycerol (TG); unlike LDL-C (non-HDL); low density lipoprotein (LDL); total cholesterol (TC); high density lipoprotein (HDL); polyunsaturated fatty acids (PUFA); eicosapentaenoic (EPA); docosahexaenoic (DHA); de novo lipogenesis (DNL);

**Table 8:** The effects of dietary fatty acids in in vivo and in vitro models with non-alcoholic fatty liver disease NAFLD.

Host	Fatty Acid Composition	Effects	References
Mice and In vitro	n-3 PUFAS - HFD-fed mice - n-3 PUFA-enriched HFD (17,18-EEQ, 5-HEPE, 9-HEPE (efficient components of HEPEs and EEQs metabolites) Duration: 4 days - In vitro: Primary hepatocytes and peritoneal macrophages	Mice: Reduced macrophage infiltration in adipose tissue - Reduced pro-inflammatory cytokines (IL-6, MCP-1 and TNF-α) in plasma content In vitro: activation of pro-inflammatory cytokines as well as activation of JNK pathway by palmitate in macrophages were reduced through the mixture of 17,18-EEQ, 5-HEPE, 9-HEPE	[123]
Mice	Corn oil and n3-PUFAs - Corn-oil based HFD - n3-PUFA DHA/EPA-enriched diet Duration: 12 weeks	- The quality of the diet (n3-PUFA) could modulate liver transcriptoma: - corn oil based HFD: modulate PPAR-related gene expression and have induced PPAR-γ gene signatures - DHA/EPA-enriched diet: induced genes known to be regulated by PPAR-α	[130]
Mice	n3-PUFAs - HFD-fed mice - n3 PUFA-enriched HFD Duration: 8 weeks	- n3-PUFA-enriched HFD: without obesity, liver damage, hypertriglyceridemia, hepatic insulin resistance, steatosis - Improved hepatic glucose output - Reduced expression of genes related to lipogenesis: SREBP-1C and FAS - Improved inflammatory markers: increase adiponectin levels - Increased beta oxidation with increased expression of PPARα and PPAR-α target and CPT-1	[131]
Mice	n3-PUFAs - HFD-fed mice - DHA/EPA supplementation in HFD (different ratios 1:2, 1:1 and 2:1) Duration: 11 weeks	- Best suggestion: Ratio 1:2 - Increase HDL/C levels - Reduced ALT, AST, MDA levels and increased glutathione (GSH) levels - Reduced the expression of lipid metabolism genes: SREPB-1C, SCD-1, ACC-1 and PPAR-γ - Lowered expression of proteins expression levels c-Jun and c-Fos - Weakened activation of Ap-1 - Reduced inflammatory cytokines (IL-6 and IL-1β)	[132]
Mice	MUFA and n3-PUFAs - Western diet supplemented with olive oil (OO) (WD + OO), - Westerm diet supplemented with EPA (WD + EPA) - Western diet supplemented with DHA (WD + DHA) - Western diet supplemented with DHA + EPA (WD + DHA/EPA) Duration: 16 weeks	- WD + OO: severe NASH phenotype accompanied with inflammation, oxidative stress and fibrosis - WD + DHA/EPA: attenuated ALT and AST levels - WD + DHA: - Reduced cell surface markers for Kupffer cells and macrophages in liver Clec4f; Clec10a; CD68; and F4/80) - Diminished inflammatory markers like IL-1β, TNF-α, TLR4, TLR-9 and genes involved in TLR pathway Cd-14 and MyD88 - Blocked WD-induced accumulation of nuclear factor κ beta (NFκB) in hepatic nuclei - Reduce oxidative stress (NADPH oxidase subunits Nox2, p22phox, p40phox, p47phox, p67phox) - Diminished Procol1α1 - Reduced cytokine TGF-β1	[133]
Mice and In vitro	MUFA and n3-PUFAs - Western diet supplemented with olive oil (OO) (WD + OO), - Westerm diet supplemented with EPA (WD + EPA) - Western diet supplemented with DHA (WD + DHA) - Western diet supplemented with DHA + EPA (WD + DHA/EPA) Duration: 16 weeks In vitro: Human LX2 stellate cells treated with DHA	WD + DHA: No increase in hepatic nuclear abundance (Smad 3) - WD+OO and WD+EPA: Increased Smad3 expression. In vitro: Human LX2 stellate cells: - Blocked TGF-β mediated induction of Col1A1	[134]
Rats	Canola Oil, Soybean Oil, Safflower Oil, Lard - High oleic canola oil (HOC) - Conventional canola oil (C) - Conventional canola oil/flax oil blend (C/F) (3:1 ratio) - High linoleic safflower oil (SF) - Soybean oil (SB) - Lard and soybean oil (L) - Weight-matched group fed lard and soybean oil (WM) Duration: 12 weeks.	- C/F group: - Attenuated hepatic stetatosis—Lower concentration of fat liver - Altered hepatic phospholipids fatty acid profile by increasing EPA and DHA. - HOC, C and C/F groups: - Gained the least of body weight: lowest weight gain without differences in adiposity	[135]
Rats	n3-PUFAs Perilla oil - High-fat diet/high-cholesterol diet (HFD/HC) - Perilla oil-enriched diet (POH)	- POH group: - Improved HFD-induced hyperlipidemia (TG, CT and LDL) - Reduced hepatic steatosis - Diminished activity of ALT and AST enzymes - Reduced hepatic inflammatory infiltration around portal area - Rescued HFD-induced hepatic fibrosis - Abrogated downregulation of ABCG 5 and ABCG 8 - Increased the expression of CYP2A1 and CYP27A1	[136]
Mice	n3-PUFAs EPA - HFD-fed mice - HFD-enriched 3% EPA + 500 mg milidronate/kg/day - HFD-enriched 3% EPA Duration: 10 days	- HFD-enriched 3%: - Accentuated hepatic triglyceride accumulation. - HFD-enriched 3% EPA + 500 mg milidronate/kg/day: - Exacerbation of milindronate-induced triglyceride accumulation - EPA decreased the milidronate-induced mRNA expression of inflammatory genes: MPEG1, COX 2, CD68, F4/80 - Increased GRP120	[137]
Mice	*n3-PUFAs and n-9 MUFAs* - Methionine and choline deficient (MCD) diet - MCD-enriched diet n-3 PUFA + n-9 MUFA (EPA/DHA 25 mg + OO 75 mg) (MCD/n-3) - MCD-enriched diet n-9 MUFA alone (OO 100 mg) (MCD/OO) two times a week by intragastric gavage. Duration: 8 weeks	- MCD/n-3 group: higher levels of ALT, severe scores of inflammation - Increased intrahepatic expression of inflammatory markers: TNF-α and CCL2 - Increased expression of profibrogenic genes: TGF-β1 - Increased tissue inhibitor of metalloproteinase (TIMP-1) - Higher portal pressure	[138]
Mice	*n-9 MUFA* - Standard chow diet (SCD) - HFD based on lard (HFD—49 energy % of fat) Duration: 12 weeks HFD-fed mice were divided in four groups: - Unchanged HFD-L (HFD-L) - HFD based on EVOO (HFD-EVOO) - HFD based on EVOO rich in phenols (HFD-OL with same percentage of fat) - R (reversion, LFD) Duration: 24 weeks	- HFD-EVOO: - Reduced body weight - Improved plasma lipid profile - Reduced pro-inflammatory citokynes in epididimal adipose tissue: IFN-γ, IL-6, leptin and macrophage infiltration - Diminished NAFLD activity (NAS) score - Reduced hepatic adiponutrin (Pnpal3) - Increased Cd36 gene	[139]
Mice and In vitro	*Palmitoleate n-7 MUFA* - LFD - LFD + Palmitoleate LFD + Oleate	- LFD+Palmitoleate: -Improved systemic insulin-sensitivity - Induced hepatic steatosis Improved insulin signaling in liver: insulin-stimulates Akt (Ser 473) phosphorylation - Reduced phosphorylation of NFκB p65 (Ser468) - Reduced expression of IL-6 and TNF-α. In vitro: hepatocytes and RAW macrophaged+palmitoleate: - Increased fat deposition’ - Stimulated FAS expression - Activated SREBP-1c - Decreased inflammation: NFκB p65 Ser 68, TNF-α, IL-6 in both hepatocytes and RAW macrophages.	[140]
In vitro	Palmitic acid (PA) SAFs In vitro: Kupffer Cells and stellate cells stimulated with TLR2 and palmitic acid	In vitro (Kupffer cells) were more important than HSC in TLR2-mediated progression of NASH - TLR 2 ligand increased NOD3 (inflammasome) in Kuppfer cells. - PA together with TLR2 ligand: Induced caspase-1 activation in Kupffer cells - Released IL-1β and IL-1α in Kuppfer cells	[141]
Rats and In vitro	Corn Oil - peroxidized Fat - Corn oil peroxidized oil (PO) - Unperoxidized FA (OIL) - Tap water (WA) gavage Duration: 6 days.	- PO group: - Increased pro-oxidant state NOS-2, NO-formation and pronounced lipid peroxidation in liver - Decrease in α- and γ-tocopherol in liver. - Increased inflammatory markers: TNFα, COX-2, IL-1β and macrophage markers cd68 and cd 163 in the liver In vitro: hepatocytes, endothelial and Kupffer cells and incubated with peroxidized linoleic acid: more pronounced in Kupffer cells: - Augmented the secretion of TNF-α, mRNA expression of TNF-α, NOS-2, COX-2 - Increased p38MAPK phosphorylation	[142]

Abbreviations: alanine aminotransferase (ALT); γ-glutamyl transpeptidase (GGT); triacylglycerol (TG); unlike LDL-C (non-HDL); low density lipoprotein (LDL); total cholesterol (TC); high density lipoprotein (HDL); polyunsaturated fatty acids (PUFA); eicosapentaenoic (EPA); docosahexaenoic (DHA); tumor necrosis factor alpha (TNF-α); monocyte chemoattractant protein-1 (MCP-1); interleukin(IL); hydroyeicosapentaenoic acid (HEPEs); cJun-N-terminal-kinase (JNK); epoxyeicosatetraenoic acid (EEQ); peroxisome proliferator-activated receptor (PPAR); Western Diet (WD); olive oil (OO); monounsaturated fatty acids (MUFA); nuclear factor κ beta (NFκB); G protein–coupled receptor 120 (GRP120); C-C motif chemokine ligand 2 (CCL-2); cicloxigenase-2 (COX-2); NO-synthetase-2 (NOS-2); p38 mitogen-activated protein kinases (p38MAPK); ATP-binding cassette hemitransporters *G5* and *G8* (ABCG 5 and 8); Cytochrome P-450 2E1 (CYP2E1); vitamin D_3_ 25-hydroxylase (CYP27A1) cDNA.
